# Does an asymmetric lobe in digital rectal examination include any risk for prostate cancer? results of 1495 biopsies

**DOI:** 10.1590/S1677-5538.IBJU.2014.0598

**Published:** 2016

**Authors:** Ömer Yilmaz, Özgür Kurul, Ferhat Ates, Hasan Soydan, Zeki Aktas

**Affiliations:** 1 GATA Haydarpasa Teaching Hospital, Department of Urology, Istanbul, Turkey

**Keywords:** Prostate, Neoplasms, Digital Rectal Examination

## Abstract

**Introduction:**

Despite the well-known findings related to malignity in DRE such as nodule and induration, asymmetry of prostatic lobes, seen relatively, were investigated in a few studies as a predictor of prostate cancer so that there is no universally expected conclusion about asymmetry. We aimed to compare cancer detection rate of normal, asymmetric or suspicious findings in DRE by using biopsy results.

**Materials and Methods:**

Data of 1495 patients underwent prostate biopsy between 2006-2014 were searched retrospectively. Biopsy indications were abnormal DRE and or elevated PSA level(>4ng/mL). DRE findings were recorded as Group 1: Benign DRE, Group 2: Asymmetry and Group 3: Nodule/induration. Age, prostatic volume, biopsy results and PSA levels were recorded.

**Results:**

Mean age, prostate volume and PSA level were 66.72, 55.98 cc and 18.61ng/ mL respectively. Overall cancer detection rate was 38.66 % (575 of 1495). PSA levels were similar in group 1 and 2 but significantly higher in group 3. Prostatic volume was similar in group 1 and 2 and significantly lower in Group 3.

Malignity detection rate of group 1,2 and 3 were 28.93%, 34.89% and 55.99% respectively. Group 1 and 2 were similar (p=0.105) but 3 had more chance for cancer detection.

**Conclusion:**

Nodule is the most important finding in DRE for cancer detection. Only an asymmetric prostate itself does not mean malignity.

## INTRODUCTION

Digital rectal examination (DRE) for prostate is an important diagnostic procedure for both benign and malignant diseases. Prostate specific antigen (PSA) and DRE are the best-known predictive factors for positive prostate biopsies (1). Although there is an increase in cancer detection with PSA, transrectal ultrasonography (TRUS) and magnetic resonance imaging (MRI) modalities, DRE is the most frequently used and the first-preferred tool for cancer detection in prostate. Indications for prostate needle biopsy (PNB) include elevated serum prostate-specific antigen (PSA) and/or abnormal DRE (2). In some studies (3), suspicious DRE findings were described as nodule, induration and asymmetry. In other studies, only induration or nodule were considered suspicious for cancer. The asymmetry, i.e. one lobe having higher volume than the other one, was defined as benign finding (4). While there are well-known findings associated with malignancy in DRE such as nodule and induration, the contour alterations or asymmetry of prostatic lobes, seen relatively, were also investigated in a few studies as predictors of prostate cancer (5, 6), so there is no universally expected conclusion regarding asymmetry.

The aim of our study was to compare the cancer detection rates of normal, asymmetric or suspicious prostate such as nodule in DRE by using TRUS guided prostate biopsy results of 1495 patients.

## MATERIALS AND METHODS

Following the approval of local ethics committee, data belonging to 1495 patients who had undergone TRUS guided tru-cut prostate biopsy in our institution between 2006 and 2014 were screened retrospectively. Biopsy indications included abnormal DRE findings such as nodule or induration identified by an urologist at our department, elevated PSA levels (>4ng/mL), increased PSA velocity (>0.7ng/mL), low free/total PSA percentage (<18%) and density. Exclusive asymmetry finding was not considered as an abnormal DRE finding for biopsy indication. Patients with asymmetric prostatic lobe had biopsy due to high PSA level or increased PSA velocity. Initial biopsies included 12 cores in most of the patients. Eighteen or 24 cores were taken from patients who had history of recurrent biopsies and larger volumes of prostate (>60cc). DRE findings were grouped as follows: group 1: patients with benign DRE, group 2: patients with asymmetric prostatic lobe, group 3: patients with nodule and/ or induration by palpation. If a lobe is found to be larger than the other in DRE, it is considered as asymmetry. The asymmetric lobes did not have any additional suspicious lesions such as nodule or induration. Age, prostatic volume on TRUS, pathology results of biopsies, and PSA levels were also recorded. Then, DRE findings, biopsy results, PSA levels and prostatic volumes of all groups were compared.

### Statistical methods

SPSS for Windows version 16.0 (SPSS Inc Chicago Illinois USA) was used for data analysis. One-Way ANOVA test and Tukey's post-hoc test were used for comparison of continuous data in multiple groups, and chi-square test was used for comparison of categorical data of any two groups. P<0.05 level was considered as significant in all analyses.

## RESULTS

Mean age of patients enrolled in this study was 66.72, mean prostatic volume on TRUS was 55.98cc and mean PSA level was 18.61ng/ mL. Overall cancer detection rate was 38.66% (575/1495) (Table-1). 819 of 1495 (54.78%) patients had benign DRE findings, 484 (32.37%) patients had suspicious DRE findings such as nodule or induration, and 192 (12.84%) patients had asymmetric lobe in DRE and asymmetry was confirmed by TRUS assessment. All asymmetric lobes in DRE had higher volume than counter lobes in TRUS assessment.

**Table 1:** Patients' data.

DRE findings	Benign	819 (54.78%)
	Asymmetry	192 (12.84%)
	Nodule/induration	484 (32.37%)
Mean age		66.72
Mean PSA (ng/dL)		18.61
Mean Prostatic volume (cc)		55.98
Mean biopsy cores		16.4
Overall cancer detection rate (%)		38.66

When we compared the groups for age, the mean age for benign and asymmetry groups was similar (p=0.607), nodule group had higher age average than benign and asymmetry groups (p=0.027, p=0.043).

PSA levels were similar in groups 1 and 2, however group 3 had significantly higher PSA levels than the others (Table-2).

**Table 2:** Mean PSA level and prostatic volume of 1495 patients.

	Benign (Group 1)	Asymmetry (Group 2)	Nodule (Group 3)	ANOVA
Mean PSA (ng/dL)	8.71±21.70	7.67±6.81	27.56±99.0	p1-2=0.973 p2-3<0.001*
Mean Prostatic Volume (cc)	56.57±29.15	59.97±31.90	53.16±32.60	p1-2=0.359 p2-3=0.027*
n	819	192	484	1495

* statistically significant

Prostatic volume was statistically similar in groups 1 and 2 (p=0.359). Group 3 had significantly lower prostatic volume compared to others (p=0.027).

We could not obtain data for asymmetric side in 16 patients (8.33%). 106 of 192 asymmetric lobes were on the right side (55.2%), and 70 were on the left side (36.45%). 67 of 192 (34.89%) patients with asymmetric lobe had cancer somewhere in whole prostate. Remaining 125 (65.10%) did not have malignancy. Asymmetric lobe included malignancy in 46 of 67 (68.65%) patients. Remaining 21 (31.34%) patients had malignancy in opposite side of asymmetry. 271 of 484 (55.99%) patients with nodule had malignancy in their prostate. Nodule and malignancy were concurrent in the same lobe in 232 of 271 patients (85.60%). 213 (44%) patients with nodule had benign results. According to biopsy results, 819 patients in total had benign DRE findings and 237 (28.93%) of them had malignancy (stage T1c).

**Figure 1:**
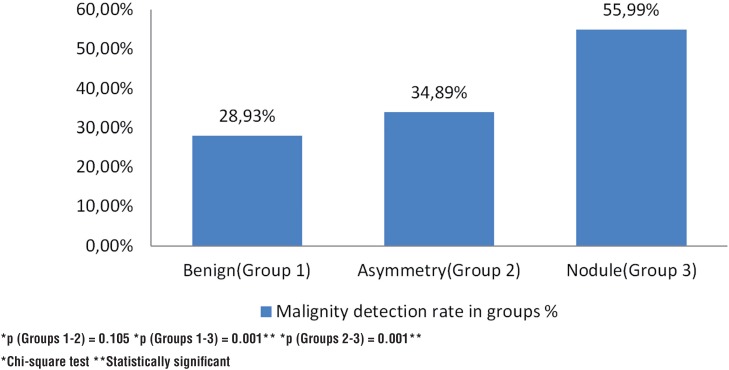
Malignancy detection rate of DRE findings.

**Figure 2:**
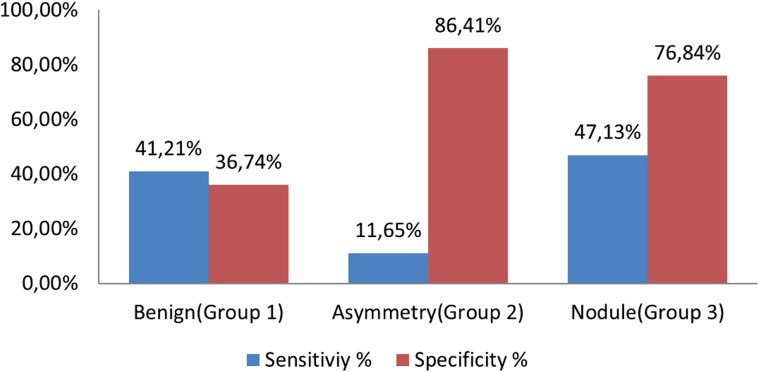
Sensitivity and specificity for cancer detection of three groups.

Malignancy detection rate for benign, asymmetry and nodule findings in DRE were 28.93%, 34.89% and 55.99% respectively. Benign and asymmetry findings were similar (p=0.105), but nodule finding had greater possibility to detect cancer compared to the other two (p=0.001) (Figure-1). Sensitivity and specificity for cancer detection for benign, asymmetry and nodule in DRE were 41.21%/36.74%, 11.65%/86.41% and 47.13%/76.84%, respectively (Figure-2).

## DISCUSSION

Despite the availability and popularity of PSA, an abnormal DRE alone is still considered an absolute indication for prostate biopsy (7). Furthermore, DRE remains an essential part of a routine physical examination, and it is useful to understand and quantify its diagnostic power for cancer detection of different findings such as benign, asymmetry and nodule. The result of DRE is usually stated as abnormal, i.e. nodularity or induration suspicious for prostate cancer, or as normal (8) the diagnostic value of different DRE findings (benign, nodule/ induration and asymmetry) as a predictor of prostate cancer has not been thoroughly evaluated in the past. Our study is the first one that evaluated the prediction value of DRE findings, especially asymmetry, in prostate cancer according to biopsy results.

Asymmetry of a prostate was defined as asymmetric growth of lateral lobes without any other suspicious findings such as nodule or induration, as assessed by DRE. Although this finding is quite often in routine prostate control, its predictive value for cancer detection is not so clear. There are only a few studies about asymmetry and its predictive value in prostate cancer in literature (5). Hansen et al. followed 963 men with no clinical evidence of prostate cancer by using PSA, and prostate biopsies were taken from men in case their PSA levels started to get increased, then asymmetry in DRE was evaluated for cancer detection among those patients they concluded that this finding was not an independent cancer predictor. Kiyoshima et al. (6) evaluated asymmetrical contours in 114 radical prostatectomy specimens and concluded that the 34% asymmetry findings were caused by cancer, and cancer-associated asymmetries showed significant correlations with aggressive signs such as cancer volume, Gleason score, positive surgical margin, and extraprostatic extension.

We evaluated the patients who had undergone prostate biopsy at our department retrospectively. Our study included all DRE findings, i.e. benign, nodule and asymmetry. Although the patients with asymmetry had somewhat more cancer detection rate than benign DRE, the difference could not reach a statistically significant level, therefore, as Hansen et al. did, we also concluded that asymmetry does not carry significant additional risk for prostate cancer. In our study, the most important and critical DRE finding for cancer detection was nodule. We only evaluated the effect of DRE findings on cancer detection of non-cancer features such as aggressiveness or extention, as biopsy results may not be enough to make such decisions.

One of the most accepted tools for prostate cancer screening and detection is PSA. When we compared the groups in terms of PSA levels, nodule group had highest correlation level with biopsy results and it was statistically significant. Although asymmetry group had higher PSA than benign group, the difference could not reach statistically significant level. PSA level findings indicated that the most important DRE finding for cancer detection is nodule, asymmetry may be deemed as benign. As prostatic volume can affect PSA level (9-11), we compared the groups in terms of prostatic volume. Benign and asymmetry group were similar according to volume but nodule group had smaller prostate significantly. It showed that detecting higher PSA level in nodule group was not influenced by prostatic volume.

It is known that prostate cancer incidence increases with advanced age (9). We also found for all groups that all of our patients who had malignancy, were older than the patients who did not have cancer. This result was consistent with PSA and biopsy results.

The cause of asymmetric growth of prostate has not been clearly understood. There may be some local factors that act differently in asymmetric lobe such as androgen receptor level or increased response to growth factors, or decreased apoptosis (12-14). In contrast with nodule (15), we cannot definitely say that cancer causes asymmetry. The most important question is whether the reason of asymmetry is malignancy or not. Although our results were not able to demonstrate that asymmetry is an apparent sign of cancer, we found that if there is malignancy in somewhere of the asymmetric prostate, the localization of this malignancy is probably in asymmetric side in an insignificant matter. Therefore, additional studies are required to come to a conclusion on the importance of asymmetry.

Normally, equal number of cores are taken from both lobes, unless suspicious areas are seen in TRUS imaging during biopsy procedure (16). That is, if a lobe is bigger than the other, more core samples may be necessary to sample both lobes equally. By doing that, the malignancy detection rate of asymmetric lobes may increase.

DRE is a subjective examination due to variability in inter-examiner findings (17). We diagnosed the asymmetry not only with DRE but also with TRUS by measuring the craniocaudal and horizontal diameters of both lobes individually. As a result, subjectivity of DRE with asymmetry was minimized. But other findings such as benign and nodule were not verified by TRUS or another examiner. This is one of the limitations of our study. Another limitation may be the retrospective nature of the study. This study only evaluated patients who underwent biopsy, meaning we do not know about patients with cancer, but have not been diagnosed with biopsy due to absence of indications such as abnormal PSA level and DRE findings. So making an exact conclusion for predictive value of DRE findings, more prospective studies may be necessary. The asymmetry in DRE has a endency to be benign based on patient's age, PSA level and biopsy result. Within our knowledge, our study is the first one that Evaluate age and PSA level in relation with cancer detection in asymmetric prostates.

## CONCLUSIONS

We found that nodule is the most important finding in DRE for cancer detection. According to our results, an asymmetric prostate itself cannot be accepted as a cancer sign. Some additional studies may be useful to come to an exact conclusion about asymmetry in prostate.
